# Co-infection of cattle with *Fasciola hepatica* or *F*. *gigantica* and *Mycobacterium bovis*: A systematic review

**DOI:** 10.1371/journal.pone.0226300

**Published:** 2019-12-30

**Authors:** Alison K. Howell, Catherine M. McCann, Francesca Wickstead, Diana J. L. Williams

**Affiliations:** 1 Department of Infection Biology, Institute of Infection and Global Health, University of Liverpool, Liverpool, United Kingdom; 2 School of Veterinary Science, University of Liverpool, Leahurst, Neston, United Kingdom; University of Illinois, UNITED STATES

## Abstract

The liver flukes, *Fasciola hepatica* and *F*. *gigantica*, are common trematode parasites of livestock. *F*. *hepatica* is known to modulate the immune response, including altering the response to co-infecting pathogens. Bovine tuberculosis (bTB), caused by *Mycobacterium bovis*, is a chronic disease which is difficult to control and is of both animal welfare and public health concern. Previous research has suggested that infection with liver fluke may affect the accuracy of the bTB skin test, but direction of the effect differs between studies. In a systematic review of the literature, all experimental and observational studies concerning co-infection with these two pathogens were sought. Data were extracted on the association between fluke infection and four measures of bTB diagnosis or pathology, namely, the bTB skin test, interferon γ test, lesion detection and culture/bacterial recovery. Of a large body of literature dating from 1950 to 2019, only thirteen studies met the inclusion criteria. These included studies of experimentally infected calves, case control studies on adult cows, cross sectional abattoir studies and a herd level study. All the studies had a medium or high risk of bias. The balance of evidence from the 13 studies included in the review suggests that liver fluke exposure was associated with either no effect or a decreased response to all of the four aspects of bTB diagnosis assessed: skin test, IFN γ, lesion detection and mycobacteria cultured or recovered. Most studies showed a small and/or non-significant effect so the clinical and practical importance of the observed effect is likely to be modest, although it could be more significant in particular groups of animals, such as dairy cattle.

## Introduction

Many parasites have the ability to modulate the host immune response in order to further their own survival. [1] This also alters the host response to co-infecting pathogens, and can have wide ranging effects, from the transmission and progression of disease to the accuracy of diagnostic tests. [2,3] The interaction between *Fasciola* spp. and *Mycobacterium bovis* is of interest in this context as both are common pathogens in cattle worldwide. [4,5]

### *Fasciola* spp.

*Fasciola hepatica* (the common liver fluke), and *F*. *gigantica* (the tropical liver fluke) are helminth parasites which have adverse effects on cattle health, welfare and production. A recent review of the literature reported that *Fasciola* spp. prevalence exceeds 60% in countries in all five continents where cattle are kept. [1] Fluke infection causes disease, subclinical effects such as reduced milk yield and growth rates, and occasionally, acute deaths. All ages of cattle are affected as protective immunity does not appear to develop. [2]

Early infections with *F*. *hepatica* are characterised by a mixed T helper (Th) 1 and 2 immune response with upregulation of interferon (IFN) γ, immunoglobulin (Ig) G1, and interleukin (IL) 4. However by 4–6 weeks after infection a Th2/regulatory T cell (Treg) response predominates, with upregulation of the cytokines IL4, IL5, IL13, transforming growth factor (TGF)β and IL10 (Flynn and Mulcahy, 2008; Gazzinelli et al., 1992; McCole et al., 1999). It is thought that fluke have evolved to induce this anti-inflammatory response in order to facilitate their own survival within the host. [6,7] Whilst the two species of fluke have a similar biology and pathology, little is known about immune responses to *F*. *gigantica* in cattle.

Diagnostic methods for fluke include faecal egg counts, antibody detection in serum or milk, and visualisation of fluke at post-mortem examination. The latter is considered the gold standard for fluke diagnosis with 100% specificity. Sensitivity varies, being up to 99% if the liver is examined thoroughly, but may be as low as 63% in a commercial abattoir setting. [8,9] Faecal egg counts (FEC) have 90–100% specificity, but sensitivity ranges from 43–80% depending on the amount of faeces used and the season. [10] Antibody-detection enzyme-linked immune-sorbent assays (ELISAs) can be used on serum and on milk samples, and have higher sensitivity than, and comparable specificity to FEC. [10,11]

### Bovine tuberculosis

Bovine tuberculosis (bTB) is caused by *M*. *bovis* and occurs in cattle throughout the world. [12] *M*. *bovis* is a slow growing intracellular bacterium with a lengthy pre-clinical phase lasting months or years.[13] To establish infection in a host, *M*. *bovis* bacilli must be taken up by macrophages.[14] A cell mediated immune response results, and a granuloma consisting of classically activated macrophages and IFN γ –producing T cells walls off the infected macrophages.[15–17] This is often sufficient to control the infection in a latent phase for many years, [13] but in some cases clinical disease develops, characterised by a drop in IFN γ and an increase in antibody levels. [18] During this phase, bacilli multiply and spread leading to disseminated granulomas, increased infectiousness, and clinical signs. [19]

In endemic countries, tuberculin skin testing is the mainstay of control programmes.[20] This entails the subcutaneous injection of tuberculin, followed by measurement of a delayed- type hypersensitivity response in the form of a lump after 72 hours.[20] Variations include injection into the tail head (used in USA, New Zealand and elsewhere)[21] and into the neck (EU).[22] In Britain and Ireland, the single intradermal comparative cervical tuberculin test (SICCT) is used, due to the high prevalence of environmental mycobacteria. For this test, an additional injection is made of avian tuberculin (PPDa) and the response compared with that of the bovine tuberculin (PPDb). Animals that show a sufficiently greater response to PPDb than PPDa are considered positive.[22] Although test specificity is high at around 99.9%, sensitivity is estimated between 50–80%. [23–26] As the test detects an antigen-specific memory T-cell response, infection is not detectable during the early stages of infection, and there can also be a lack of response during advanced disease due to a predominant humoral response. [13,23,26] Immune responses can also be suppressed by factors such as corticosteroid treatment, parturition and production related stress.[26]

The Bovigam^®^ TB Kit (Thermo Fisher Scientific Inc., MA, USA) which measures IFN γ response is also used in some situations, usually as a more sensitive but less specific test in bTB positive herds.[20] Again, this test compares responses to PPDa and PPDb. Positive animals (known as ‘reactors’) to any test are either retested or compulsorily slaughtered and attempts are made to confirm bTB infection by lesion detection, histopathology and culture. Post-mortem diagnosis is by finding lesions in affected tissues, most commonly the lungs and lymph nodes, and by culture and/or histopathology of tissues to confirm the diagnosis.[20] No gold standard for the detection of bTB exists, with all available tests having a relatively poor sensitivity.[26] Not all endemic countries have control programmes, particularly where resources are limited.[27]

### Co-infection with *Fasciola* spp. and *M*. *bovis*

There has long been a concern that liver fluke may affect the outcome of the bTB skin test, and that this may hamper control programmes for bTB. The primary aim of this review was to examine the evidence to determine the effect of liver fluke infection on four outcomes relevant to bTB diagnosis: SICCT (or other similar diagnostic skin test), IFN γ test, lesion detection and bacterial culture. The secondary aim was to try to understand why different study approaches may have led to opposing outcomes.

## Materials and methods

The PRISMA guidelines for systematic reviews from the Cochrane Collaboration were followed [28]. A protocol (S1 File) and PRISMA check list (S2 File) can be found in the supporting information.

### Type of studies

All types of study were considered for inclusion, as long as they included animals co-infected with liver fluke and tuberculosis, plus a control group testing negative for liver fluke. We searched for studies on *F*. *hepatica*, *F*. *gigantica*, *M*. *bovis* and *M*. *tuberculosis*, in any species of host. Observational studies and experimental studies were considered.

### Fluke measures

Any method of herd or individual fluke diagnosis was considered.

### TB measures

The outcomes of interest were TB diagnostic measures, whether pre- or post-mortem. Some studies had looked at various other measures of immune response, but for the purposes of this study, we only included those measures that were related to diagnosis: SICCT response, IFN γ test, lesion detection, culture or bacterial recovery, and other commercially available tests such as antibody assays.

### Search methods

**Fig 1:**
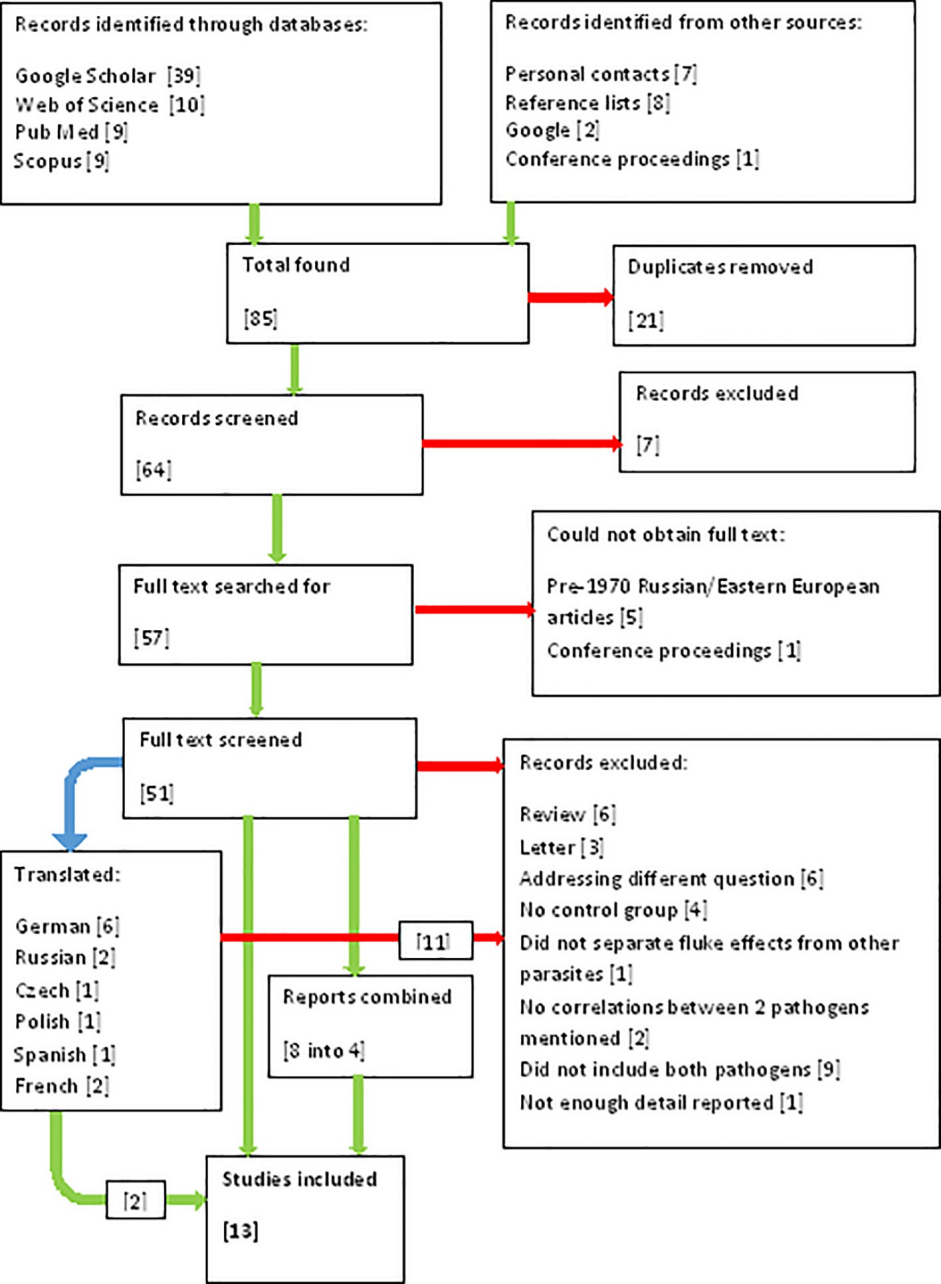
Numbers of studies found and removed at each stage of the systematic review (PRISMA flow diagram).

Searches were carried out in Google Scholar, Scopus, Web of Science and Pub Med using combinations of the following search terms: Fasciola, liver fluke, tuberculosis, tuberculin, mycobacterium, M. bovis and BCG. Searching was performed separately by two researchers. Further searches were carried out in Google and in the conference proceedings of the World Association for the Advancement of Veterinary Parasitology, Society for Epidemiology and Preventive Veterinary Medicine, the International *Mycobacterium bovis* Conference, International Conference of Parasitology and British Society for Parasitology. Hand searches of reference lists from recovered papers were performed. Finally, personal contacts from other research institutions were approached to ask for any unpublished studies. See Fig 1 for details of studies found and removed at each stage.

### Data collection and analysis

#### Selection of studies

Search results were merged and duplicates removed. Titles and abstracts were examined and obviously irrelevant papers removed. The full text of reports was obtained where possible, through the British Library or by contacting the main research institutions in the countries of origin. For the English language papers, these were all read independently by two researchers initially. For foreign language studies, these were initially screened by one researcher able to read the language. Where papers were considered to be of interest, another colleague was sought for a second opinion.

Papers were discarded at this stage according to the following criteria: a review or letter rather than an original study, a different research question addressed, no control group, did not separate fluke effect from other parasites, did not mention co-infections between the two parasites, or did not include both pathogens.

### Data extraction and management

Studies were read in detail by at least two researchers to assess the quality of evidence and extract the data (data extraction forms were piloted before use, S3 File). Effect size and direction, statistical significance and author interpretation of the findings were recorded. In the case of disagreement, a consensus was reached by discussion or by seeking a third opinion. Studies were numbered in order of date of publication. Where a single study was reported in more than one place, the reports were grouped together and given a single number. A small number of studies were rejected at this stage due to inability to extract useful data.

### Assessment of the risk of bias

The risk of bias was categorised as low, medium or high for each study, based on consideration of the following:

#### Study design

Low: Randomised experiment i.e. a study where a randomly selected proportion of individuals are exposed in a standardised way to the pathogens of interest

High: All other study designs including observational studies: cohort, case control and cross sectional designs.

#### Randomisation (Applicable only to experimental studies)

Low: Animals were randomly allocated to a control or intervention (i.e. exposure to relevant pathogen) group, with a suitable method of randomisation.

High: No appropriate method of randomisation was reported, or randomisation was not used

#### Sampling bias

Low: Animals were a representative sample from the population

Medium: Selection of animals was not related to the outcomes of interest, however animals came from a source that meant only a part of the underlying population was represented

High: Study design made bias likely, e.g. animals were selected based on features related to the outcomes of interest

#### Comparability of exposed and non-exposed animals

Low: Exposed and non-exposed animals (i.e. all the animals in the study whether considered positive or negative for the pathogens) were drawn from the same underlying population, and the most important confounders were controlled for in the analysis. For case control studies, matching on confounders is an alternative option. The main confounders were considered to be age, breed, type (dairy or beef), herd size, region or factors associated with region such as climate variables and the prevalence of bTB in the area [29–32].

Medium: Some but not all confounders were controlled for

High: Exposed and non-exposed animals were not from the same population, and/or confounders not controlled for.

#### Detection bias

Low: The same diagnostic tests were used for all groups of animals

High: The diagnostic test used was determined by infection status or treatment group, or it was implemented differently between the groups.

#### Blinding

Low: The report mentioned that there was blinding at all of the following stages: administration of intervention (if possible), data collection, diagnoses or measurements taken, laboratory work, and statistical analysis

High: There was no mention of blinding being used at one or more of these stages

#### Incomplete outcome data

This refers to animals that were selected to be part of the study but for which some or all results were not reported.

Low: All data were complete, or reasons for incomplete data were analysed and found to be unlikely to introduce bias (i.e. for reasons unrelated to the infection status of the animals)

High: Incomplete information for a proportion of study subjects with no explanation, or for reasons likely to introduce bias

#### Selective reporting

Low: All aspects of the study that were mentioned in the methods were fully reported in the results. Interpretation of results and discussion of the implications fitted what the results showed

High: Some procedures that were mentioned in the methods were missing from the results. Results were over interpreted or claims were made that were not supported by the results.

### Data synthesis

As the studies were heterogenous in their methods and outcome measures, as well as being few in number, a meta-analysis was not possible. Instead a harvest plot and narrative synthesis was used to summarise the results [33].

## Results

### Results of search

A total of 85 reports were identified: 67 through database searches, eight through hand searching reference lists of other papers, seven through personal contacts, two through searching the internet using Google, and one from conference proceedings. Full details of the sources of the studies can be found in Fig 1. Following removal of duplicates and initial screening, 57 reports remained. These were published between 1950 and 2019 and were published in seven languages.

Six studies were excluded because the full text could not be obtained. Five of these were Russian or Eastern European studies dating from the 1950s-70s. One study was omitted because it was reported in conference proceedings with insufficient details. See S4 File for a list of the excluded studies. The full text was screened for 51 studies.

### Included studies

Thirteen studies were included in the final analysis, and are summarised in Table 1. Eleven were published in peer reviewed journals, often in more than one paper, and part of one study has not been published. One was a government report with part of the study reported as a poster at a conference and one study was reported in a PhD thesis. Their publication dates ranged from 1962 to 2019.

**Table 1:** A summary of the studies meeting the criteria for inclusion in the systematic review of bovine tuberculosis (bTB) and liver fluke.

Study	Reported in	Sample size	Country	Type of study	bTB outcome measure	Fluke outcome measure	Findings
1	Schanzel and Stolarik, 1962 [34]	10,711	Czechoslovakia	Cross-sectional abattoir study with PM 1, general abattoir cattle population, single abattoir	Skin test (B) Lesion presence (B)	Gross evidence of liver fluke infection (B)	1. Fluke infected cattle less likely to have false negative bTB skin test (significant, *p* = 0.02) 2. Fluke infected cattle less likely to have lesions (significant, *p* = 0.003, only if controlling for skin test result)
2	Meyer, 1963 [35]	320	EastGermany	Cross-sectional abattoir study with PM 1, general abattoir cattle population, single abattoir	Skin test (B) Lesion presence (B)	Gross evidence of liver fluke infection (B)	1. No difference in the chance of false negative bTB skin test in fluke-infected compared to fluke-free cattle 2. Fluke infected cattle less likely to have lesions (significant, *p* = 0.03)
3	Broughan et al., 2008; DEFRA, 2005 [36,37]	400	UK	Case control study with 200 bTB reactors, 200 in contacts (partially matched but not from same farm). Beef and dairy cattle, PM3	Culture-confirmed lesion (B) SICCT (B)	Liver fluke antibodies (B)	Cattle with fluke antibodies less likely to have confirmed bTB in both SICCT positive and negative animals. Significant in dairy reactors only, *p* = 0.005
4	Flynn et al., 2007 [38]	18	Ireland	Calves, experimentally infected with BCG and/or fluke. 4 groups: Fluke only, Fluke first then BCG 4 weeks later, BCG then fluke 4 weeks later, and BCG only. SICCT carried out 13w after BCG infection. PM3 after 23 weeks	SICCT (B) IFN γ (B)	Antibody ELISA (Q) Liver fluke numbers (Q)	1. Co-infected calves more likely to test negative on both Bovigam^®^ test and SICCT than BCG only–in both the BCG first and the fluke first groups. 2. No difference in IFN γ response to bPPD 3. Fluke only calves had higher fluke numbers than co-infected, and fluke-BCG had more fluke than BCG-fluke (non-significant) 4. No difference in fluke ELISA between bTB infected and uninfected
5	Flynn et al., 2009; Garza-Cuartero et al., 2016, unpublished (Jim McNair, personal communication) [39,40]	18	Northern Ireland	Calves, experimentally infected in 3 groups: fluke only, fluke and *M*. *bovis*, and *M*. *bovis* only. PM3 after 14 weeks	SICCT (B) IFN γ (Q) Antibody ELISA (B) Lesion size (Q) Lesion count (Q) Culture-positive tissues (Q) *M*. *bovis* bacterial recovery (Q) Lesion quality (D)	Antibody ELISA (B) Liver fluke numbers (Q)	1. Fluke only calves had higher fluke numbers than co-infected (non-significant) 2. Co-infected had lower IFN γ production than *M*. *bovis* only (significant only at certain time points) 3. Co-infected group had fewer bTB lesions (non-significant) 4. Co-infected group had fewer culture positive lesions 5. Bacterial recovery was lower in co-infected (significant) 6. Fewer SICCT positives in the co-infecteds than in *M*. *bovis* only (non-significant) 7. No difference in fluke ELISA between bTB infected and uninfected
6	Munyeme et al., 2012 [41]	1680	Zambia*(Fasciola gigantica)*	Cross sectional abattoir study with PM1 followed by culture. General abattoir cattle population across 13 abattoirs	Unclear: either lesion presence or culture-confirmed lesion presence (B)	Current infection (B)	Cattle infected with fluke were significantly more likely to have bTB lesions
7	Claridge, 2012 [42]	80	UK	Case control. 40 matched pairs of lactating dairy cattle, 20 each of reactors and inconclusive reactors.	SICCT (B; positive either R or IR)	Antibody ELISA (Q)	No difference between groups in terms of fluke antibody levels, *p* = 0.5
8	Claridge et al., 2012 [43]	3026 herds	UK	Cross sectional dairy herd level study using bulk milk tank samples and herd bTB status.	Herd SICCT breakdown (B)	Antibody ELISA (Q; smoothed)	1. Positive fluke test is significant negative predictor for bTB breakdown 2. Fluke antibodies and bTB breakdown are spatially separated
9	Claridge et al., 2012a; Garza-Cuartero et al., 2016 [39,43]	12	Northern Ireland	Calves, experimentally infected in 2 groups: *M*. *bovis* only and fluke and *M*. *bovis*. SICCT after 10 and 21 weeks. PM3 after 22 weeks	SICCT (Q) IFN γ (Q) Lesion size (Q) Lesion count (Q) Culture-positive tissues (Q) *M*. *bovis* bacterial recovery (Q) Lesion quality (D)	Antibody ELISA (B) Liver fluke numbers (Q)	1. Co-infected had smaller response to SICCT (although no change to qualitative result i.e. all still reactors)(significant, *p*≤0.05) 2. *M*. *bovis* bacterial load lower in co-infected (significant, *p*≤0.05) 3. No difference in lesions between groups 4. IFN γ response lower in co-infected (significant only at certain time points)
10	Byrne et al., 2017 and Byrne et al., 2018 [44,45]	6242/5698	Northern Ireland	Cross sectional abattoir study with PM2, SICCT positive and SICCT negative in-contacts cattle only, single abattoir	SICCT reaction size (Q) Lesion count (Q) Max. lesion size (Q) Lesion presence (B)	Current infection (B) Liver damage (B) Either (B)	1. No difference in bTB lesion presence between liver fluke and non liver fluke groups (current or previous fluke infection) 2. No difference in SICCT reaction size 3. No difference in number of lesions 4. Maximum lesion size smaller in fluke infected (n = 2471)
11	Kelly et al., 2018 [46]	732	Cameroon *(Fasciola gigantica)*	Cross sectional abattoir study with PM1, general slaughter population, single abattoir. Fulani and mixed breed cattle	Lesion presence (B) IFN γ PPDa-PPDb (Q) Bovigam^®^ test (B)	Liver damage (B)	1. Co-infected animals more likely to have bTB lesion [mixed breed] 2. Co-infected animals more likely to have false negative IFN test [mixed breed] 3. Fulani cattle more likely to have bTB lesions than mixed breed
12	Byrne et al., 2019a [47]	138,566	Northern Ireland	Cross sectional abattoir study with PM 1/2 followed by culture. Single abattoir. Populations examined include all cattle slaughtered at abattoir, LRS, cNRs or reactors	SICCT (B; standard and severe analysed separately) Lesions (B) Culture confirmation (B) SICCT reaction size (Q)	Active liver fluke infection and/or fluke damage (B)	1. No association between liver fluke and SICCT result 2. SICCT reaction size smaller in fluke infected cattle (non-significant) 3. No association between liver fluke and lesion detection 4. No association between liver fluke and bTB confirmation 5. Liver fluke infected animals less likely to have false negative SICCT (could indicate fewer lesions rather than effect on SICCT)
13	Byrne et al., 2019b [48]	1494 herds	Northern Ireland	Dairy herd level study. Repeat bulk milk samples	Culture-confirmed herd breakdown (B) SICCT herd breakdown (B) Lesions at routine slaughter (B) Herd breakdown size (Q)	Antibody ELISA (B and Q)	Possible small size effects but near universal liver fluke infection could have hidden the result

Q indicates quantitative or ordinal measure. B indicates binary measure. D indicates description of differences

PM1: Routine post-mortem examination that is routinely carried out in the slaughterhouse for all cattle. PM2: Standard PM for reactors involves examination of more tissues than that done routinely, but is still carried out according to the usual protocols followed by abattoir staff. PM3: Detailed PM has been carried out by researchers and may include more in depth examination e.g. slicing and soaking the liver

Three studies used experimentally infected calves, and were performed by the same research group in Ireland. BCG was used to infect calves in one experiment (study 4) and *M*. *bovis* was used in the other two (studies 5 and 9). The latter two experiments were reported in three different papers, see Table 2 for details. The design of the three studies was similar. The SICCT and interferon gamma levels were compared between calves co-infected with *M*. *bovis* and *F*. *hepatica* and singly infected calves a number of weeks post infection. In the *M*. *bovis* studies, presence of lesions and recovery of mycobacteria from tissues were also measured.

**Table 2:** A summary of where the outcomes from studies 5, 9 and 10 were published.

Allocated study number	Aspect of study	Reported in
5	SICCT	Unpublished
	Lesions	[40]
	Bacterial recovery	[39]
	IFN γ	[40]
9	SICCT	[43]
	Lesions	[39]
	Bacterial recovery	[39]
	IFN γ	[39]
10	Presence of visible lesions	[44]
	Tuberculin reaction size, post-mortem lesion counts, pathology	[45]

A case control study looking at the association between fluke antibody levels of individual adult cattle and their bTB test result was reported in a PhD thesis (study 7). Studies 8 and 13 investigated the association between bTB herd breakdown and the fluke antibody level in bulk milk tank samples from the same herd. These were all UK studies.

There were five cross-sectional abattoir studies, from Czechoslovakia (study 1), Germany (study 2), Zambia (study 6), UK (study 12), and Cameroon (study 11). The Zambian and Cameroonian studies were on the tropical liver fluke *F*. *gigantica*, and investigated the association between bTB lesions and presence of fluke in the liver at slaughter. The Cameroon study also looked at Bovigam^®^ test results. The other three studies investigated the association between bTB skin test result and presence of bTB lesions at the abattoir, comparing cattle with and without evidence of liver fluke infection (studies 1, 2 and 12).

Study 10 was an in depth project using different subsets of the same population of Northern Irish cattle slaughtered for bTB control, to look at associations between liver fluke (determined by post-mortem evidence) and different aspects of bTB including presence of lesions, lesion counts, SICCT reaction size and bacteriological confirmation. Most of the included cattle were slaughtered due to positive SICCT results (cases), with a small proportion being negative ‘in contact’ cattle (controls). The findings were written up in two papers (see Table 2 for details).

Finally, there was a case control study carried out by DEFRA in the UK (study 9). This was a large study looking at many aspects of bTB infection in cattle, of which liver fluke was one small part. The population comprised SICCT reactors and ‘in contacts’ (defined as cattle which had been in contact with bTB reactors but were not from the same farm as the bTB reactors used in the study). Fluke exposure was measured by antibody ELISA and bTB was defined as lesions confirmed by either culture or histology [36]. Some aspects of this study were written up in a poster [37].

### Risk of bias in included studies

#### Study design

Of the thirteen studies considered, three were laboratory studies, and the remaining ten were observational studies, of which two were case control studies, and eight were cross sectional studies.

#### Sampling bias

For studies 1, 2, 6, 11 and 12, abattoir populations were used. These may be geographically biased and may also be more limited in terms of age and general health than the underlying population: for example, most cattle of beef breeds are likely to be young and in good condition whereas those of dairy breeds may be older.

Studies 3 and 10 did not include any TB free cattle–only those that either tested positive or were in contact with positive cattle. These studies are aimed specifically to look at the potential for under diagnosis in cattle considered to be high risk.

Studies 8 and 13 used dairy herds only. Study 7 used lactating cattle. Therefore only adult female cattle of dairy breeds were included.

Studies 4, 5 and 9 were experimental studies which used immature cattle.

Convenience sampling was used for all of the observational studies, and random sampling is not a realistic option for observational studies of this nature. All of the studies have attempted to sample representatively from the populations that they target. The underlying population may affect the generalisability of results, especially as one study found marked differences between dairy and beef cattle.

#### Randomisation

None of the experimental trials specified a method of randomisation.

#### Blinding

Many of the study designs led to blinding at certain points, for example the SICCT administrator would generally be unaware of the animal’s fluke status, but none of the studies mentioned blinding at other stages.

#### Comparability of exposed and unexposed animals

In all observational studies, exposed and non-exposed animals were drawn from the same underlying population. All of the main confounders of age, breed, sex and region were adjusted for by including in regression models in studies 10 and 12. Of the two herd-level studies, study 13 controlled for region whilst study 8 did not. Studies 3 and 11 controlled for three of the main confounders. Study 6 controlled only for region of origin. In study 7, cases and controls were matched by farm, which would include factors including herd size, region of origin and TB history of the farm, but not of the age of the animal. Studies 1 and 2 did not control for any confounders. This measure did not apply to the experimental studies (4,5 and 9) as the small numbers of animals included were all of a similar age, sex and breed and kept under controlled conditions. Table 3 shows the confounders adjusted for in each study.

**Table 3:** The confounders controlled for in each study included in the systematic review on liver fluke and bovine TB.

Study	Age	Breed	Sex	Region	Others
1	no	no	no	no	
2	no	no	no	no	
3	yes	yes	no	yes	Flukicide treatment, herd size, season, test interval
4	NA	NA	NA	NA	
5	NA	NA	NA	NA	
6	no	no	no	yes	
7	no	no	no	no	
8	NA	NA	NA	no	Herd size, environmental/climate factors
9	NA	NA	NA	NA	
10	yes	yes	yes	yes	Herd of origin
11	yes	yes	yes	no	
12	yes	Herd type (dairy or non-dairy)	yes	yes	Herd size, year, environmental/climate factors
13	NA	NA	NA	yes	Herd size, bTB history in herd and locality, season

#### Detection bias

Study 4 had unexplained differences between sampling times between the different groups of calves. In this study, there were two co-infected groups. These groups were both administered the two pathogens 4 weeks apart, but in different orders. The *M*. *bovis* only group and the *M*. *bovis* then *F*. *hepatica* group were sampled for IFN γ at weeks 1, 3, 5 and 13 after BCG infection whereas the *F*. *hepatica* then *M*. *bovis* co-infected group were only sampled at weeks 1 and 13. The changes observed in IFN γ level mostly occurred at weeks 3 and 5, so would not have been detected in the *F*. *hepatica* then *M*. *bovis* group even if they had occurred.

#### Incomplete outcome data

Study 6 reported missing data on the region of origin of the cattle which could have introduced bias. Studies 10, 11, 12 and 13 reported missing data for logistical reasons which are unlikely to have caused bias.

#### Selective reporting

Studies 5 and 9 did not report qualitative (positive/negative) Bovigam^®^ results although use of the test was reported. Only the quantitative result of the IFN γ response was shown. There was a statistically significant difference between the mean IFN γ results of the groups at 3 out of 8 (study 5) or 2 out of 15 time points (study 9), yet the authors interpreted that this was likely to affect the outcome of diagnostic tests. The difference in skin measurements between *M*. *bovis* infected groups of calves with and without *F*. *hepatica* for study 5 was not statistically significantly different, and has never been published. Taken together, these findings suggest a reporting bias.

Study 11 describes culture methods and regression analysis for these data, but the results are not provided. The proportions of the culture are given in another paper [49] but modelling results not described.

#### Other sources of bias

Study 8 used smoothed fluke ELISA PP values for each farm as independent variables in a logistic regression model. Smoothing is a statistical process which aims to capture patterns in data whilst reducing noise. However, artificially reducing variability which may be due to genuine differences in fluke exposure on different farms can cause inflation of regression co-efficients, artificially enhancing the observed effect of fluke exposure. Studies 4, 5, 8 and 9 were done by the same group of collaborating authors and some also on the same calves, increasing the risk of confirmation bias, as authors who have previously reported one result are probably more likely to publish similar findings in the future. Studies 10, 12 and 13 were also done by a single group.

#### Summary of potential for bias

None of the studies met the required criteria for avoiding bias. This is not surprising for the observational studies, but even for the experimental studies, missing information in both the methods and results made it difficult to interpret the validity of the findings.

Experimental studies may provide more consistent evidence due to similarity of infectious doses and the animals used, whereas in naturally infected animals it may be difficult to be sure whether an animal is infected at all. In the three laboratory studies included here, the infectious doses of the pathogens and routes of infection were similar to those animals might experience in the field [50]. However, experiments are generally designed to maximise the chance of finding an effect by infecting animals with each pathogen in a particular order and measuring outcomes at optimal time points. Table 4 summarises the assessments of bias for each study.

**Table 4:** Summary of bias for the included studies.

Study number	Study design	Sampling bias	Random allocation	Blinding	Comparability of groups	Detection bias	Incomplete outcome data	Selective reporting
1			NA					
2			NA					
3			NA					
4					NA			
5					NA			
6			NA					
7			NA					
8			NA					
9					NA			
10			NA					
11			NA					
12			NA					
13			NA					

Red denotes a high risk of bias, yellow, medium, and green, a low risk of bias. NA (not applicable) refers to measures which do not apply to the study due to its design.

### Narrative synthesis

#### Skin test

Eleven studies investigated the effect of fluke on the SICCT or other tuberculin skin test. Studies 4, 5 and 9 found that the response to the SICCT was reduced in fluke-infected calves. Study 4 had the largest effect, with 4/5 BCG-only infected calves testing positive whilst 1/9 co-infected calves tested positive. Study 4 also investigated the relative timing of the infections, and found that the greatest effect was seen when the animal was infected with *F*. *hepatica* before BCG, but the effect was still observed when the animal was infected with BCG first. Studies 5 and 9 had similar experimental designs, using virulent *M*. *bovis* to infect the calves. In study 5 (unpublished results, Jim McNair personal communication), 6/6 of the *M*. *bovis*-only calves tested positive for bTB compared to only 4/6 of the co-infected calves. In study 9, all *M*. *bovis* only and co-infected calves tested positive but there was a significantly greater reaction in the *M*. *bovis* only group compared to the co-infected group (raw skin measurements unpublished but differences between avian and bovine reactions published in [43]).

Similar to study 9, study 12 found a reduction in SICCT reaction size that did not affect the binary test result. Studies 2, 7, 10 and 13 found no effect. All were observational studies. Studies 10, 12 and 13 were rigorous, detailed studies and adjusted for all main confounders. Interestingly, significant associations were found in univariable analysis which then disappeared in the multivariable analysis, highlighting the importance of adjusting for confounders. Study 10 compared SICCT positive cattle with SICCT negative in-contacts, a group which is considered high risk for having undetected bTB. Study 12 looked at the entire abattoir population and subdivided them into SICCT reactors at either standard or severe interpretation. Study 13 was a herd level study and 93% of herds had fluke exposure, which could have restricted the ability to detect a difference between fluke infected and uninfected. A further limitation of herd level studies is that fluke infection levels vary widely between individuals and usually only a very small proportion of animals test positive for bTB at any herd breakdown; we cannot tell whether the same individuals within the herd are at risk from each disease. Study 2 had a high risk of bias as no confounders were controlled for. Study 7 was under-powered and 95% of cattle had fluke, again limiting the ability to detect a difference.

Study 8 showed that fluke infected herds were one third less likely to have a cow test positive for bTB on the skin test. However, region was not adjusted for in the model, and the smoothing of explanatory variables may have inflated the effect size. In addition, herd-level study limitations apply as for study 13.

Study 3 was a case control study with bTB reactors and in contact animals (a similar approach to study 10). Fluke was found to be a significant negative predictor for confirmed bTB (by lesion/culture/histology) in both SICCT test reactors and non-reactors, but only in dairy animals. The authors interpreted these findings as fluke causing false positives to the SICCT. However, an alternative explanation more consistent with the evidence from other studies would be that fluke decreases likelihood of finding visible lesions.

The data from study 1 were used to determine that cattle with fluke had a decreased chance of a false negative skin test. However, the data showed that the skin test had close to 100% sensitivity and specificity in fluke positive cattle, which suggests a problem in the dataset somewhere.

Overall, the evidence points towards fluke infection being associated with a reduced response to the SICCT, although this effect is unlikely to be large enough to be of clinical significance in naturally infected adult cattle on farms.

#### Interferon γ

Four studies looked at interferon γ, studies 4, 5 and 9 under experimental conditions and study 11 in Cameroon. Study 4 reported both Bovigam^®^ qualitative results (positive/negative) and the quantitative IFN γ response to PPDb stimulation for individuals. However, some of those reported as testing positive using Bovigam^®^ had lower IFN γ levels than those reported as testing negative. This was because the response to PPDa was higher in the co-infected animals (R. Flynn, personal communication), but this was not seen in any other study, so could be an artefact. In studies 5 and 9 the mean IFN γ was consistently higher in the *M*. *bovis* group than in the co-infected group, but this is a small difference and is only statistically significant at a small number of time points. The statistical test used was not detailed in either of the reports. Study 11 found a statistically significant difference between cattle with and without fluke pathology, in cattle positive by *M*. *bovis* culture. However, the majority of results from both groups were still classed as negative indicating unreliable test performance in this setting. In a separate analysis of cattle with negative IFN γ test, there was increased risk of a false positive in mixed breed cattle with fluke infection, however, this did not apply to Fulani breed cattle. Overall the evidence suggests that fluke is associated with a decreased response to the Bovigam^®^ test, but this may be too small to be clinically important.

#### Lesions

Nine studies reported on lesions. In experimentally infected animals, study 5 reported lower numbers of lesions in co-infected animals compared to *M*. *bovis* only, although this was not a statistically significant difference, whilst study 9 found no difference between the groups. Observational studies 10, 12 and 13 also found no effect.

The authors of studies 1 and 2 (cross-sectional abattoir studies) considered that the differences between groups were too small to be clinically significant. However, our analysis of the data provided in the papers showed a significant decrease in lesions in fluke-infected cattle in both studies, although confounders could not be adjusted for meaning the risk of bias is high.

Studies 10, 12 and 13, all large scale observational studies, found no effect after adjusting for covariates.

Study 6, also a cross sectional study, reported that fluke infected cattle were five times more likely to have bTB lesions than those without fluke. This is a much greater effect size than seen in the other studies. This study took place in Zambia, where *F*. *gigantica* is the endemic species of fluke, and there is no routine bTB testing programme, so there could be important differences in management and the stages of bTB occurring in the animals. There were also some errors and inconsistencies in the analysis and reporting which cast doubt on the reliability of the results. Findings from study 11, also in a tropical setting where there is no routine testing, supported study 6 findings, but only in mixed breed cattle.

Overall the evidence does not support hypothesis that liver fluke has an effect on bTB lesion detection, although if the two African studies are excluded, there is some support for the theory of decreased visible lesions in fluke infected animals.

#### Culture

Five studies describe culture/bacterial recovery. Studies 5 and 9 were experimental studies, and all lung and lymph node tissues with or without lesions were examined, rather than culturing only from lesions as would be the case in the UK bTB control programme or most abattoir studies. The results of both studies were analysed together and there was a significant difference, however in study 5, only one *M*. *bovis*-only infected animal actually had a greater number of bacteria and the other *M*. *bovis* only and co-infected animals all had similar amounts. The difference in bacterial recovery between fluke infected and fluke negative groups was more marked in study 9, which could have been because these calves were slaughtered at 22 weeks post-infection compared to 14 weeks in study 5. There were only six calves per group in each study and the data were skewed with most calves having low numbers of bacteria. Studies 12 and 13, after adjusting for confounders, found no effect. Overall, the evidence points towards a decrease in the ability to culture *M*. *bovis* from lesions in fluke infected animals. However, this effect is not as clear in naturally infected cattle.

#### Summary

**Fig 2:**
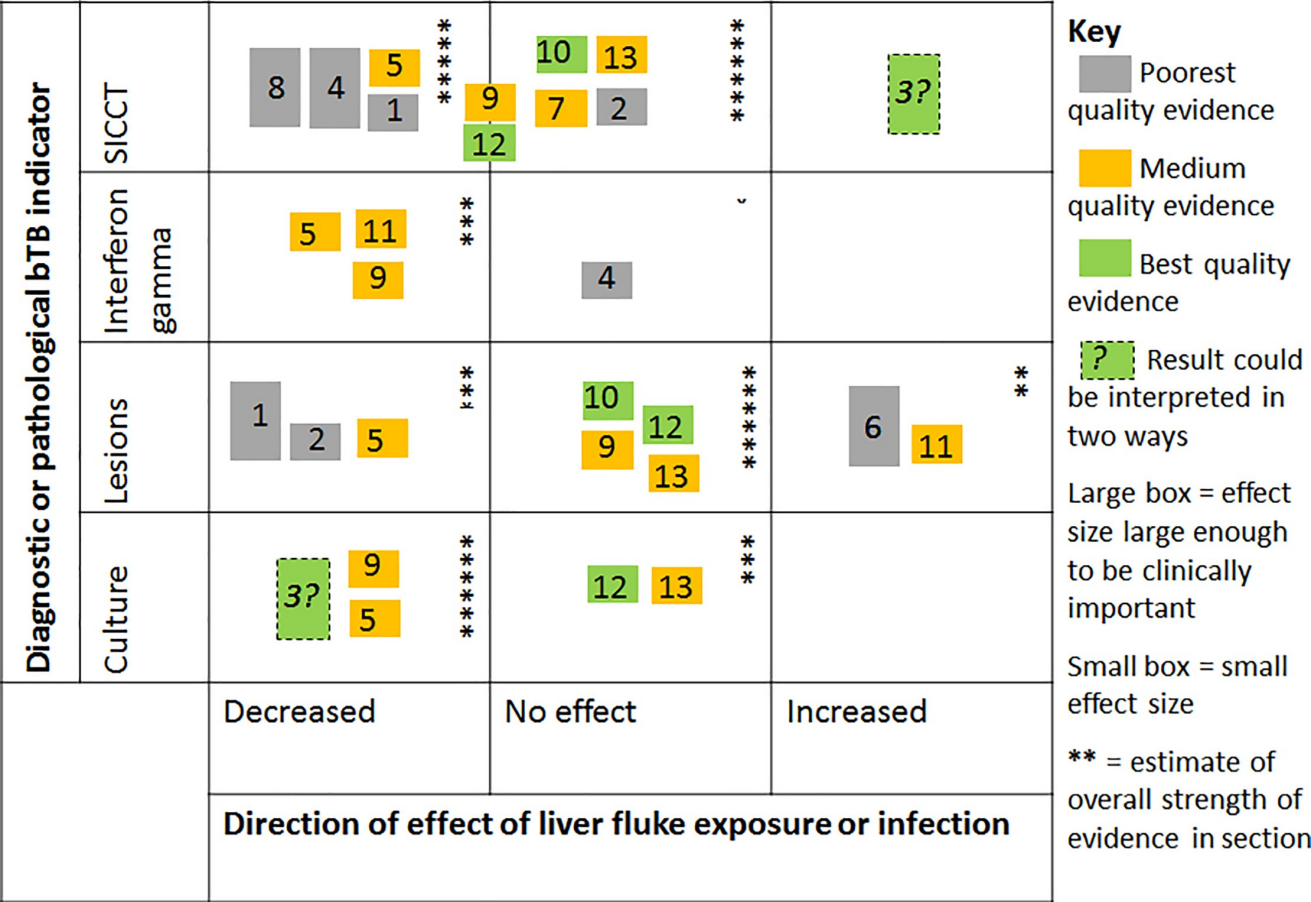
A harvest plot showing the results from the thirteen studies included in the analysis. The numbers correspond to study numbers given in Tables 1 and 3. Studies which cover more than one aspect are included more than once. Quality of evidence relates to the likelihood of bias and the clarity of reporting. The size of the box is an assessment of the likely clinical importance of the finding, if it were true. Small effect boxes include some results considered statistically significant. The number of stars was decided by allocating a value of 2 for best quality evidence, 1 for medium and 0.5 for poorest quality evidence, and multiplying this by 1 for a small box and 2 for a large box, then summing all the values within that section. Studies are shown on the border line if there was a difference in skin test reaction size but this did not affect binary skin test result.

Overall, most of the studies found that liver fluke exposure was associated with either no effect or a decreased response to all of the four aspects of bTB diagnosis assessed: skin test, IFN γ, lesion detection and mycobacteria cultured or recovered (Fig 2). Most of the studies found a small effect. A decrease was more likely to be seen in experimental studies than observational studies. Those showing the largest effects were generally those where the evidence was deemed of poorer quality.

## Discussion

This is the first systematic review on co-infection with *M*. *bovis* and *Fasciola* spp. and its impact on bTB diagnosis. Studies were included if they contained sufficient data to contribute evidence on the association between liver fluke infection and four measures relating to bTB diagnosis. Due to the paucity of studies on co-infection with *M*. *bovis* and *Fasciola* spp., we wished to maximise the amount of information obtained by including both observational and experimental studies. Although observational studies do not usually meet strict criteria for avoidance of bias, they provide information about co-infection in naturally infected animals under conditions that are difficult to replicate in the laboratory. This led to the inclusion of studies of widely varying design, which made direct comparison of results difficult, and precluded doing a meta-analysis. A harvest plot was used instead to synthesise the data and aid in drawing conclusions. Both the quality of the evidence and the clinical importance of the effect size were taken into account. The use of this semi-quantitative method gave a representation of the strength of the evidence for each measure, but was necessarily somewhat subjective. The balance of evidence from the thirteen studies included in this review supports the hypothesis that liver fluke-infected animals are likely to have a reduced response to both the SICCT (or other tuberculin skin test) and the Bovigam^®^ test and fewer bacteria recovered/cultured from their lesions. The clinical and practical importance of this effect is likely to be small, and many studies particularly observational studies of naturally infected cattle showed no effect. The main body of evidence showed no effect of liver fluke infection on visible lesions detection.

Differing findings between studies can arise by chance but may also be due to study design. In all three of the studies on experimentally infected animals, the effect of liver fluke infection was to reduce the response to the skin test. However, four studies on naturally infected cattle found no association, and one study possibly found the opposite effect (depending on interpretation) [36]. There is more variability in field studies, which could obscure small effects. In addition, interpretation is made more difficult by not knowing whether the absence of lesions is due to the animal not being infected or because it did not produce any detectable lesions in spite of infection. A number of studies were excluded from the review because they were designed to test the hypothesis that fluke caused false positives on the SICCT, and therefore did not include bTB infected animals. However, several of these studies showed that fluke infected animals did not have false positives on the bTB skin test [51–54]. It seems likely that, in the absence of a gold standard test for bTB, so-called ‘false positives’ to the SICCT seen in populations where bTB is endemic are actually due to a lack of lesions rather than the absence of bTB infection.

The lower risk of bias in experimental studies, combined with work showing that fluke infection down-regulates Th1 type immune responses [5,55] supports the view that fluke infection can reduce the size of the response to the SICCT. However, the effect size in the two experimental studies that used *M*. *bovis* rather than BCG was small in terms of SICCT test interpretation. This leads to the conclusion that the effect size is probably small, and could explain why it was not seen in four of the 11 observational studies. Similarly, all three of the studies on experimentally infected animals that looked at IFN γ levels found a small but consistent reduction in co-infected animals. It is doubtful that this effect would be large enough to affect Bovigam^®^ test outcomes. There was one study of the Bovigam^®^ test in naturally infected animals, which supports these results in that there was a difference but it was only seen in a small proportion of animals.

Of studies that looked at lesions and culture/bacterial recovery, the picture was more varied in both experimentally and naturally infected animals. One explanation for the reduction in lesion observation and/or culture/bacterial recovery reported in fluke infected cattle in many of the studies is that an interaction is mediated via the host immune system. In fluke infected cattle, macrophage phenotype switching occurs with alternatively activated macrophages becoming the predominant type. These are less efficient at phagocytosis and this reduces the rate of uptake of bacilli [39], which could lead to fluke infected cattle being more resistant to infection. However, logically, if fluke infected cattle do take up *M*. *bovis* bacilli, the altered immune environment with a reduced Th1-type response could lead to less efficient granuloma formation, increasing mycobacterial dissemination around the body, but making it less likely that lesions will be seen at post-mortem. This raises the possibility of a non-linear relationship between the two pathogens, with the observed association depending on factors such as the order in which an individual is exposed, the length of time between infections, how long the animal has been infected for, and the infectious dose. Post-mortem examination can reveal current and/or past liver fluke infections. However, no difference in association with bTB was found between the two metrics in studies 10 and 12. Study 7 [38] reported that the effect size was greater for cattle that were infected with *F*. *hepatica* before BCG, than for cattle which were infected in the opposite order. Associations due to immune interaction may be more obvious in experimentally infected animals, where natural variability between individual animals’ immune responses can be reduced by using a homogenous group of cattle, and timing and dose of infection can be standardised.

Also of interest was that in two studies where fluke numbers were counted, there was a consistent although non-significant finding that co-infected animals had fewer flukes at post-mortem than those infected with *F*. *hepatica* only [38,40]. This applied regardless of the order of infection with the two pathogens. The possibility that bTB could be protective against fluke infection should not be ruled out.

A shortcoming of most of the studies reviewed here is that, due to either the difficulties of keeping cattle under laboratory conditions, or the compulsory bTB control programme, studies only looked at relatively early stage bTB infections. In fact, the two studies (6 and 11) that found a positive correlation between bTB lesions and liver fluke infection were both undertaken in settings where there is no routine test and slaughter programme, meaning that bTB could have reached a more advanced stage in some animals in the studies. A possible explanation for this finding is therefore that, once bTB is established, fluke infection could accelerate progression towards clinical tuberculosis. In general, a non-linear relationship between *F*. *hepatica* and *M*. *bovis* infections could lead to inconsistent findings unless the pathogens are measured quantitatively.

Correlations may also be observed that are not due to direct interactions between the two pathogens, and this is more likely in naturally infected animals due to the non-random selection of which animals become infected. Animals have inherent varying resistance to parasites, and this resistance has a cost [56]. Therefore, animals whose immune responses make them relatively resistant to parasites could be more prone to contracting other pathogens. For example, studies of nematodes and bTB in buffaloes found that those that were more resistant to nematodes were more susceptible to bTB, so the two pathogens were inversely correlated [57]. Conversely, animals that were susceptible to nematodes but were treated to reduce their burdens suffered less morbidity and mortality as a result of bTB, compared to animals from the same population that were not treated [58]. If this same effect was true of fluke, flukicide treatment could confound the findings if not adjusted for in observational studies of naturally infected animals.

A review of co-infection studies in wild animals found that cross sectional studies can find associations that are in the opposite direction to the underlying interaction [59]. The effects of co-infection are context- specific and may only be important under certain conditions relating to the relative timings, burdens of infection, or in certain population groups such as old, young, pregnant, metabolically stressed [13,59]. This could explain the differences between the studies in this review, and also the differences between dairy and beef cattle seen in study 3 [36], where there was a very large difference in effect size between beef and dairy cattle. The interaction may also be of greater importance in contexts where the infections are able to progress further than would occur in a managed setting, for example in wild or feral populations.

The quality of a systematic review is limited by the available evidence. There was a medium to high risk of bias in all of the studies included in this review. To some extent this cannot be avoided with observational studies, however, certain measures could be taken in order to ensure the data are analysed fairly, without the temptation to try to extract statistically significant results. We suggest that for future studies, choosing the statistical methods to be used before data collection begins would help to reduce bias arising at the analysis stage, as would keeping the statistician unaware of the disease status of the animals. There was evidence of author bias in the different conclusions reached from similar results: in study 9 [43], the authors inferred that a reduction in size of reaction to PPDb would cause marked under detection of bTB even though this reduction was not sufficient in magnitude to change the result of the SICCT. Conversely, the authors of study 12 [47], who similarly found a reduction in reaction size that did not affect the outcome of the SICCT, inferred that this was not clinically important. Another example is that of study 4 [37] who concluded that unconfirmed SICCT reactors were false positives rather than bTB positive animals with atypical lesions. These examples illustrate the difficulty in disentangling the true meaning of study findings and highlight the value of a systematic review of the data.

Studies 10, 12 and 13 [44,45,47,48] were undertaken in an effort to follow up the previous findings and used large sample sizes and numerous modelling approaches to try to identify any possible association between the two pathogens. This collection of studies was the most thorough in controlling for confounders and the most rigorous in reporting all aspects of the study. Interestingly there are several examples of association between liver fluke infection and bTB on univariable analysis that disappeared in the multivariable analysis. This illustrates the importance of circumspect judgement of results from any observational study that does not control adequately for the main confounders.

The authors of some of the included studies have collaborated with the research group at the University of Liverpool. This meant that we were able to obtain some extra unpublished information which improved our understanding of these studies. This is likely to have improved the review by enabling us to include more complete data. Conversely, due to the difficulties associated with obtaining and reading some of the older foreign language studies, and the fact that methods were reported very briefly and authors were not contactable, some studies had to be excluded. This may have biased the conclusions reached in this analysis.

In summary, there is evidence than liver fluke infection may have an effect on the diagnosis of bTB by both skin test and Bovigam^®^. Although it is likely that the practical importance of this effect is small, the possibility of greater effects in particular sub groups of animals, such as older or dairy cattle, should be considered. This begs the question of whether liver fluke should be considered an impediment to the eradication of bTB. [60–62] Despite extensive testing and culling, bTB incidence in England, Wales and Northern Ireland has risen consistently over recent years, with the situation in Ireland not much better.[63] A recent review discussed possible reasons why attempts at controlling bTB in these countries have been so much less successful than in continental Europe, and posited the relatively high prevalence of liver fluke as a possibility.[64] This is pertinent as liver fluke is becoming more common in this region due to climate change.[65] Whilst there are still questions to be answered, particularly around the relative timings of the infections, the current data suggests that, with limited resources, bTB control efforts should probably be focused on other factors.

## Supporting information

S1 File Systematic review protocol. (DOCX)

S2 File PRISMA checklist. (DOC)

S3 File Data extraction form. (DOCX)

S4 File List of excluded studies on co-infection with liver fluke and bTB. (DOCX)

## References

[1] Mehmood K, Zhang H, Sabir AJ, Abbas RZ, Ijaz M, Durrani AZ, et al A review on epidemiology, global prevalence and economical losses of fasciolosis in ruminants. Microb Pathog. 2017;109: 253–262. 10.1016/j.micpath.2017.06.006 28602837

[2] Clery D, Torgerson P, Mulcahy G. Immune responses of chronically infected adult cattle to Fasciola hepatica. Vet Parasitol. 1996;62: 71–82. 10.1016/0304-4017(95)00858-6 8638395

[3] Gazzinelli R, Oswald I, James S, Sher A. IL-10 inhibits parasite killing and nitrogen oxide production by IFN-gamma-activated macrophages. J Immunol. 1992;148: 1792–6. Available: http://www.jimmunol.org/content/148/6/1792.short 1541819

[4] Flynn RJ, Mulcahy G. The roles of IL-10 and TGF-beta in controlling IL-4 and IFN-gamma production during experimental Fasciola hepatica infection. Int J Parasitol. 2008;38: 1673–80. 10.1016/j.ijpara.2008.05.008 18597757

[5] McCole DF, Doherty ML, Baird AW, Davies WC, McGill K, Torgerson PR. T cell subset involvement in immune responses to Fasciola hepatica infection in cattle. Parasite Immunol. 1999;21: 1–8. 10.1046/j.1365-3024.1999.00188.x 10081766

[6] Altmann DM. Review series on helminths, immune modulation and the hygiene hypothesis: nematode coevolution with adaptive immunity, regulatory networks and the growth of inflammatory diseases. Immunology. 2009;126: 1–2. 10.1111/j.1365-2567.2008.03006.x 19120492 PMC2632705

[7] Moreau E, Chauvin A. Immunity against Helminths: Interactions with the Host and the Intercurrent Infections. J Biomed Biotechnol. 2010; 10.1155/2010/428593 20150967 PMC2817558

[8] Rapsch C, Schweizer G, Grimm F, Kohler L, Bauer C, Deplazes P, et al Estimating the true prevalence of Fasciola hepatica in cattle slaughtered in Switzerland in the absence of an absolute diagnostic test. Int J Parasitol. 2006;36: 1153–1158. 10.1016/j.ijpara.2006.06.001 16843470

[9] Mazeri S, Sargison N, Kelly RF, Bronsvoort BM., Handel I, Brennan G. Evaluation of the performance of five diagnostic tests for Fasciola hepatica infection in naturally infected cattle using a Bayesian no gold standard approach. Yu X, editor. PLoS One. CABI publishing; 2016;11: e0161621 10.1371/journal.pone.0161621 27564546 PMC5001639

[10] Charlier J, De Meulemeester L, Claerebout E, Williams D, Vercruysse J. Qualitative and quantitative evaluation of coprological and serological techniques for the diagnosis of fasciolosis in cattle. Vet Parasitol. 2008;153: 44–51. 10.1016/j.vetpar.2008.01.035 18329811

[11] Salimi-Bejestani MR, McGarry JW, Felstead S, Ortiz P, Akca A, Williams DJL. Development of an antibody-detection ELISA for Fasciola hepatica and its evaluation against a commercially available test. Res Vet Sci. 2005;78: 177–181. 10.1016/j.rvsc.2004.08.005 15563926

[12] O’Reilly LM, Daborn CJ. The epidemiology of Mycobacterium bovis infections in animals and man: a review. Tuber Lung Dis. 1995;76 Suppl 1: 1–46. Available: http://www.ncbi.nlm.nih.gov/pubmed/7579326 10.1016/0962-8479(95)90591-x 7579326

[13] Pollock JM, Neill SD. Mycobacterium bovis infection and tuberculosis in cattle. Vet J. 2002;163: 115–127. 10.1053/tvjl.2001.0655 12093187

[14] Podinovskaia M, Lee W, Caldwell S, Russell DG. Infection of macrophages with Mycobacterium tuberculosis induces global modifications to phagosomal function. Cell Microbiol. 2013;15: 843–59. 10.1111/cmi.12092 23253353 PMC3620910

[15] Flynn JL, Chan J, Triebold KJ, Dalton DK, Stewart TA, Bloom BR. An essential role for interferon gamma in resistance to Mycobacterium tuberculosis infection. J Exp Med. 1993;178: 2249–54. Available: http://www.ncbi.nlm.nih.gov/pubmed/7504064 10.1084/jem.178.6.2249 7504064 PMC2191274

[16] Tufariello JM, Chan J, Flynn JL. Latent tuberculosis: mechanisms of host and bacillus that contribute to persistent infection. LANCET Infect Dis. 2003;3: 578–590. Available: http://infection.thelancet.com 10.1016/s1473-3099(03)00741-2 12954564

[17] McGill JL, Sacco RE, Baldwin CL, Telfer JC, Palmer M V, Waters WR. The role of gamma delta T cells in immunity to Mycobacterium bovis infection in cattle. Vet Immunol Immunopathol. 2014;159: 133–43. 10.1016/j.vetimm.2014.02.010 24636303

[18] Welsh MD, Cunningham RT, Corbett DM, Girvin RM, McNair J, Skuce RA, et al Influence of pathological progression on the balance between cellular and humoral immune responses in bovine tuberculosis. Immunology. Blackwell Science Ltd; 2005;114: 101–111. 10.1111/j.1365-2567.2004.02003.x 15606800 PMC1782060

[19] Liebana E, Johnson L, Gough J, Durr P, Jahans K, Clifton-Hadley R, et al Pathology of naturally occurring bovine tuberculosis in England and Wales. Vet J. 2008;176: 354–360. 10.1016/j.tvjl.2007.07.001 17728162

[20] Anon. Bovine tuberculosis. OIE Terrestrial Manual. OIE; 2018. Available: https://www.oie.int/fileadmin/Home/eng/Health_standards/tahm/3.04.06_BOVINE_TB.pdf

[21] Anon. USDA APHIS | Tuberculosis [Internet]. 2017 [cited 27 Sep 2019]. Available: https://www.aphis.usda.gov/aphis/ourfocus/animalhealth/nvap/NVAP-Reference-Guide/Control-and-Eradication/Tuberculosis

[22] Anon. EU Council Directive on animal health problems affecting intra-Community trade in bovines and swine [Internet]. 1964 [cited 27 Sep 2019]. Available: https://eur-lex.europa.eu/legal-content/EN/TXT/PDF/?uri=CELEX:01964L0432-20150527&rid=1

[23] de la Rua-Domenech R, Goodchild AT, Vordermeier HM, Hewinson RG, Christiansen KH, Clifton-Hadley RS. Ante mortem diagnosis of tuberculosis in cattle: A review of the tuberculin tests, gamma-interferon assay and other ancillary diagnostic techniques. Res Vet Sci. 2006;81: 190–210. 10.1016/j.rvsc.2005.11.005 16513150

[24] Karolemeas K, McKinley TJ, Clifton-Hadley RS, Goodchild A V, Mitchell A, Johnston WT, et al Recurrence of bovine tuberculosis breakdowns in Great Britain: Risk factors and prediction. Prev Vet Med. 2011;102: 22–29. 10.1016/j.prevetmed.2011.06.004 21767886

[25] Neill SD, Bryson DG, Pollock JM. Pathogenesis of tuberculosis in cattle. Tuberculosis. 2001;81: 79–86. 10.1054/tube.2000.0279 11463227

[26] Strain SAJ, McNair J, McDowell SWJ. Bovine tuberculosis: A review of diagnostic tests for M. bovis infection in cattle [Internet]. 2011. Available: https://www.daera-ni.gov.uk/sites/default/files/publications/dard/afbi-literature-review-tb-review-diagnostic-tests-cattle.pdf

[27] Anon. Bovine tuberculosis: OIE—World Organisation for Animal Health [Internet]. [cited 27 Sep 2019]. Available: https://www.oie.int/en/animal-health-in-the-world/animal-diseases/bovine-tuberculosis/

[28] The Cochrane Collaboration. Cochrane Handbook for Systematic Reviews of Interventions [Internet]. Version 5. Higgins J, Green S, editors. 2011. Available: www.handbook.cochrane.org

[29] Bennema SC, Ducheyne E, Vercruysse J, Claerebout E, Hendrickx G, Charlier J. Relative importance of management, meteorological and environmental factors in the spatial distribution of Fasciola hepatica in dairy cattle in a temperate climate zone. Int J Parasitol. 2011;41: 225–233. 10.1016/j.ijpara.2010.09.003 20887726

[30] Olsen A, Frankena K, Bødker R’, Toft N, Thamsborg SM, Enemark HL, et al Prevalence, risk factors and spatial analysis of liver fluke infections in Danish cattle herds. 2011; 10.1186/s13071-015-0773-x 25888827 PMC4374337

[31] Bessell PR, Orton R, White PCL, Hutchings MR, Kao RR. Risk factors for bovine Tuberculosis at the national level in Great Britain. BMC Vet Res. 2012;8: 51 10.1186/1746-6148-8-51 22564214 PMC3406951

[32] Skuce RA, Allen AR, McDowell SWJ. Herd-level risk factors for bovine tuberculosis: a literature review. Vet Med Int. 2012;2012: 621210 10.1155/2012/621210 22966479 PMC3395266

[33] Ogilvie D, Fayter D, Petticrew M, Sowden A, Thomas S, Whitehead M, et al The harvest plot: A method for synthesising evidence about the differential effects of interventions. BMC Med Res Methodol. BioMed Central; 2008;8: 8 10.1186/1471-2288-8-8 18298827 PMC2270283

[34] Schanzel H, Stolarik L. The influence of liver flukes on the specificity of the intradermal tuberculin test in cattle. Vet Med Praha. 1962;7: 39.

[35] Meyer W. Hetero-allergy to the tuberculin reaction in cattle. Monatsh Veterinarmed. 1963;18: 325–328.

[36] DEFRA. Report on TB SE3013. 2005.

[37] Broughan JM, Durr P, Clifton-Hadley R, Colloff A, Goodchild T, Sayers R, et al. Bovine tuberculosis and Fasciola hepatica infection. Proceedings of the Society of Veterinary Epidemiology and Preventive Medicine Conference 2009. London; 2009. Available: http://www.svepm.org.uk/posters/2009/Broughan; Bovine tuberculosis and Fasciola hepatica infection.pdf

[38] Flynn RJ, Mannion C, Golden O, Hacariz O, Mulcahy G. Experimental Fasciola hepatica Infection Alters Responses to Tests Used for Diagnosis of Bovine Tuberculosis. Infect Immun. 2007;75: 1373–1381. 10.1128/IAI.01445-06 17194810 PMC1828587

[39] Garza-Cuartero L, Blanco A, Mcnair J, Flynn RJ, Williams D, Diggle P, et al Fasciola hepatica Infection Reduces Mycobacterium and Mycobacterial Uptake Suppresses the Pro-inflammatory Response. Parasite Immunol. 2016;38: 387–402. 10.1111/pim.12326 27108767 PMC6680181

[40] Flynn RJ, Mulcahy G, Welsh M, Cassidy JP, Corbett D, Milligan C, et al Co-Infection of Cattle with Fasciola hepatica and Mycobacterium bovis—Immunological Consequences. Transbound Emerg Dis. 2009;56: 269–274. 10.1111/j.1865-1682.2009.01075.x 19575746

[41] Munyeme M, Munang’andu HM, Nambota A, Muma JB, Phiri AM, Nalubamba KS. The Nexus between Bovine Tuberculosis and Fasciolosis Infections in Cattle of the Kafue Basin Ecosystem in Zambia: Implications on Abattoir Surveillance. Vet Med Int. 2012;2012: 921869 10.1155/2012/921869 23213629 PMC3504483

[42] Claridge JA. Does Fasciola hepatica infection increase the susceptibility of cattle to infection with other pathogens normally controlled by a Th1 or pro-inflammatory response? This thesis has been submitted in accordance with the requirements of the University of Li. 2012;

[43] Claridge J, Diggle P, McCann CM, Mulcahy G, Flynn R, McNair J, et al Fasciola hepatica is associated with the failure to detect bovine tuberculosis in dairy cattle. Nat Commun. 2012;3 10.1038/ncomms1840 22617293 PMC3989536

[44] Byrne AW, Graham J, Brown C, Donaghy A, Guelbenzu-Gonzalo M, McNair J, et al Bovine tuberculosis visible lesions in cattle culled during herd breakdowns: the effects of individual characteristics, trade movement and co-infection. BMC Vet Res. BioMed Central; 2017;13: 400 10.1186/s12917-017-1321-z 29284483 PMC5747088

[45] Byrne AW, Graham J, Brown C, Donaghy A, Guelbenzu-Gonzalo M, McNair J, et al Modelling the variation in skin-test tuberculin reactions, post-mortem lesion counts and case pathology in tuberculosis-exposed cattle: Effects of animal characteristics, histories and co-infection. Transbound Emerg Dis. Wiley/Blackwell (10.1111); 2018;65: 844–858. 10.1111/tbed.12814 29363285

[46] Kelly RF, Callaby R, Egbe NF, Williams DJL, Victor NN, Tanya VN, et al Association of Fasciola gigantica Co-infection With Bovine Tuberculosis Infection and Diagnosis in a Naturally Infected Cattle Population in Africa. Front Vet Sci. Frontiers; 2018;5: 214 10.3389/fvets.2018.00214 30238010 PMC6136300

[47] Byrne AW, McBride S, Graham J, Laheurta-Marin A, McNair J, Skuce R, et al Liver fluke (Fasciola hepatica) co-infection with bovine tuberculosis (bTB) in cattle: a retrospective animal-level assessment of bTB risk in dairy and beef cattle. Transbound Emerg Dis. 2019; 10.1111/tbed.13083 30484969

[48] Byrne AW, Graham J, McConville J, Milne G, Guelbenzu-Gonzalo M, McDowell S. Liver fluke (Fasciola hepatica) co-infection with bovine tuberculosis (bTB) in cattle: a prospective herd-level assessment of herd bTB risk in dairy enterprises. Transbound Emerg Dis. 2019; 10.1111/tbed.13209 31012527

[49] Egbe NF, Muwonge A, Ndip L, Kelly RF, Sander M, Tanya V, et al Abattoir-based estimates of mycobacterial infections in Cameroon. Nat Sci Reports. 2016;6 10.1038/srep24320 27075056 PMC4830956

[50] Dean GS, Rhodes SG, Coad M, Whelan AO, Cockle PJ, Clifford DJ, et al Minimum infective dose of Mycobacterium bovis in cattle. Infect Immun. American Society for Microbiology; 2005;73: 6467–6471. 10.1128/IAI.73.10.6467-6471.2005 16177318 PMC1230957

[51] Kokurichev PI, Karabainov MA. Specificity of the tuberculin test in cattle with fascioliasis. Sb Nauchnikh Tr Leningr Inst Usovershenstvovaniya Vet Vrachei. 1957;11: 81–85. Available: http://www.cabdirect.org/abstracts/19620800018.html?freeview=true

[52] Manukyan ZK. Non-specific tuberculin reactions in cattle with fascioliasis. Tr Armyanskogo Nauchno-Issledovatelskogo Vet Instituta. 1955;8: 25–28. Available: http://www.cabdirect.org/abstracts/19550800674.html

[53] El-Ahwal AMA. [Effect of experimental fascioliasis on the results of the intradermal tuberculin test in the guinea pig]. Berliner und Münchener tierärztliche Wochenschrift. 1969;82: 484–5. Available: http://europepmc.org/abstract/med/5399020 5399020

[54] Hartwigt H, El-Ahwal AMA. Untersuchungen ueber die bedeutung der Fasciolose als Ursache positiver Tuberkulinreaktionen beim Rind. Berliner und Muenchener Tieraerztliche Wochenschrift1. 1968;81: 315–316.

[55] Bossaert K, Farnir F, Leclipteux T, Protz M, Lonneux JF, Losson B. Humoral immune response in calves to single-dose, trickle and challenge infections with Fasciola hepatica. Vet Parasitol. 2000;87: 103–23. Available: http://www.ncbi.nlm.nih.gov/pubmed/10622602 10.1016/s0304-4017(99)00177-6 10622602

[56] Hayward AD, Garnier R, Watt KA, Pilkington JG, Grenfell BT, Matthews JB, et al Heritable, Heterogeneous, and Costly Resistance of Sheep against Nematodes and Potential Feedbacks to Epidemiological Dynamics. Am Nat. 2014;184: S58–S76. 10.1086/676929 25061678

[57] Jolles AE, Ezenwa VO, Etienne RS, Turner WC, Olff H. Interactions between macroparasites and microparasites drive infection patterns in free-ranging African buffalo. Ecology. 2008;89: 2239–2250. 10.1890/07-0995.1 18724734

[58] Ezenwa VO, Jolles AE. Opposite effects of anthelmintic treatment on microbial infection at individual versus population scales. Science (80-). 2014;347: 175–7. 10.1126/science.1261714 25574023

[59] Fenton A, Knowles SCL, Petchey OL, Pedersen AB. The reliability of observational approaches for detecting interspecific parasite interactions: Comparison with experimental results. Int J Parasitol. Australian Society for Parasitology Inc.; 2014;44: 437–445. 10.1016/j.ijpara.2014.03.001 24704058

[60] Howell A, Baylis M, Smith R, Pinchbeck G, Williams D. Epidemiology and impact of Fasciola hepatica exposure in high-yielding dairy herds. Prev Vet Med. Elsevier B.V.; 2015;121: 41–48. 10.1016/j.prevetmed.2015.05.013 26093971 PMC4528078

[61] McCann CM, Baylis M, Williams DJL. Seroprevalence and spatial distribution of Fasciola hepatica-infected dairy herds in England and Wales. Vet Rec. BioMed Central; 2010;166: 612–617. 10.1136/vr.b4836 20472872

[62] Bloemhoff Y, Forbes A, Danaher M, Good B, Morgan E, Mulcahy G, et al Determining the Prevalence and Seasonality of Fasciola hepatica in Pasture-based Dairy herds in Ireland using a Bulk Tank Milk ELISA. Ir Vet J. 2015;68: 16 10.1186/s13620-015-0042-5 26157575 PMC4495626

[63] Abernethy DA, Upton P, Higgins IM, McGrath G, Goodchild A V, Rolfe SJ, et al Bovine tuberculosis trends in the UK and the Republic of Ireland, 1995–2010. Vet Rec. 2013;172 10.1136/vr.100969 23292950

[64] Allen AR, Skuce RA, Byrne AW. Bovine Tuberculosis in Britain and Ireland–A Perfect Storm? the Confluence of Potential Ecological and Epidemiological Impediments to Controlling a Chronic Infectious Disease. Front Vet Sci. Frontiers; 2018;5: 109 10.3389/fvets.2018.00109 29951489 PMC6008655

[65] Caminade C, Van Dijk J, Baylis M, Williams D. Modelling recent and future climatic suitability for fasciolosis in Europe. Geospat Health. 2015;9: 301 10.4081/gh.2015.352 25826311

