# Supplementary material for: Co-infection of cattle with Fasciola hepatica or F. gigantica and Mycobacterium bovis: A systematic review
Source: PLoS One. 2019 Dec 30;14(12):e0226300. doi: 10.1371/journal.pone.0226300 (PMC6936813; doi:10.1371/journal.pone.0226300)
Supplement: S1 File — (DOCX) [file pone.0226300.s001.docx]

**Systematic review protocol: liver fluke and TB co-infection**

**Objective**

To determine the effect of liver fluke infection on outcomes relevant to bTB diagnosis. Also, to investigate why different studies find opposing outcomes.

**Specific definitions**

| Population of interest | Expect that most studies will concern domestic cattle (any age, any breed, any country, any use), but if studies about humans or other species are discovered these will be considered for inclusion |
| --- | --- |
| Liver fluke infection | *Fasciola hepatica* or *gigantica*. As determined by faecal egg count, post mortem, antibody detection or copro-antigen detection |
| tuberculosis | Confirmed disease by post mortem exam/culture, or suspected disease diagnosed by SICCT, Interferon gamma test |
| Type of study | Any – either observational or intervention study |
| Inclusion criteria | Any primary research study looking at co-infection with tuberculosis and liver fluke |
| Exclusion criteria | No useful data obtainable |
| Search terms | Combinations of: Fasciola, liver fluke, tuberculosis, tuberculin, mycobacterium, M. bovis and BCG. |
| Search strategy  (2 people) | Search databases : Google Scholar, Scopus, Web of Science, PubMed  Grey literature (Conference proceedings: WAAVP, SVEPM, M bovis conference, BSP)  Hand searches of reference lists of papers found  Search online for relevant DEFRA documents  Ask personal contacts |
| Select studies (2 people) | Merge search results to remove duplicates  Examine title/abstract to remove obviously irrelevant studies. If in doubt keep in  [Keep records of all papers found and which discarded at which stage]  Retrieve full text of potentially relevant reports  Assign a number to each study and to each paper (work out if any studies have been reported more than once in different places and link together)  Examine full reports to see if meet eligibility criteria  May need to contact authors at this stage  Discuss and reach consensus  Make final list of included papers for further analysis |
| Assess methodology (2 people) | Use standardised form (pilot and revise as necessary): Type of study, risk of bias, confounding, quality of study  Discuss and reach consensus  Contact authors for further info if needed |
| Extract data (2 people) | Use standardised form (pilot and revise as necessary)  Study population, method of measuring outcome and intervention, effect size plus precision, statistical significance, author conclusions  Record source of funding and conflict of interest  Contact authors for further info if needed  Discuss and reach consensus |
| Analysis | If sufficient studies are found to do a meta-analysis, RCTs will be analysed separately from observational studies.  Assess publication bias  Description, tables, harvest plots etc  Consider possible reasons for any discrepancy: difference between experimental/field conditions, age of animals, differences in measurement of exposure/outcome, bias, confounding, etc |
