# Supplementary material for: Co-infection of cattle with Fasciola hepatica or F. gigantica and Mycobacterium bovis: A systematic review
Source: PLoS One. 2019 Dec 30;14(12):e0226300. doi: 10.1371/journal.pone.0226300 (PMC6936813; doi:10.1371/journal.pone.0226300)
Supplement: S3 File — (DOCX) [file pone.0226300.s003.docx]

**Bovine TB and Liver Fluke Systematic Review**

| Study ID | Author(s) | Year |
| --- | --- | --- |
| Report ID | Reviewed by | Date |

**Methods:**

| Age |  |
| --- | --- |
| Number |  |
| Study population |  |
| Selection method |  |
| Duration |  |
| Setting |  |
| Country |  |
| Fluke detection methods |  |
| TB detection methods |  |
| Details of intervention/dose rate etc |  |
| Design | Retrospective/prospective  Data source of retrospective |

**Risk of bias**

| Random allocation (RCT only)  Yes | No |
| --- | --- |
| Allocation concealment (RCT only)  Yes | No |
| Selection/sampling bias  No | Yes/possible |
| Response bias (questionnaire studies)  No | Yes/possible |
| Recall bias (questionnaire studies)  No | Yes/possible |
| Comparability of groups assessed?  How? | No  Groups materially different |
| Detection bias (different tests used for different groups)  No | Yes/possible |
| Confounders considered?  Which ones?  [Age, herd size, region, | No |
| Blinding  Yes (Who, At which stage of study)  No but probably not causing bias | No and may cause bias |
| Missing data:  None  Present, accounted for, not causing bias | Present but not accounted for  Present, accounted for causing bias or unclear  Not mentioned |
| All methods performed and reported as described | Some results missing or analysed differently to methods |
| Overall risk of bias  Low | High |
| Quality of study  High | Low |
|  |  |

**Analysis:**

Described fully?

**Results:**

Data (size and direction of any differences, statistical significance)

Author conclusions:

Funding source

References to be followed up:

Author correspondence required

Any other comments
