# Supplementary material for: Co-infection of cattle with Fasciola hepatica or F. gigantica and Mycobacterium bovis: A systematic review
Source: PLoS One. 2019 Dec 30;14(12):e0226300. doi: 10.1371/journal.pone.0226300 (PMC6936813; doi:10.1371/journal.pone.0226300)
Supplement: S4 File — (DOCX) [file pone.0226300.s004.docx]

### List of excluded published studies on co-infection with liver fluke and bTB

Abdul-Fattah MM, El-Motayam M, El-Shami EA, Salem GA, Soliman AM, Khorshed SE. Effect of Toxoplasma co-infected with intestinal helminths on cell mediated immunity to tuberculosis patients. J Egypt Soc Parasitol. 2008;38: 895–902.

Aleksandrov NA. [Specificity of the tuberculin test in fascioliasis and echinococcosis]. Veterinariia. 1969;10: 45–6.

Ameni G, Medhin G. Effect of Gastro-intestinal Parasitosis on Tuberculin Test for the Diagnosis of Bovine Tuberculosis. J Appl Anim Res. 2000;18: 221–224. doi:10.1080/09712119.2000.9706347

Anishchenko AK. Influence of fascioliasis on the tuberculin test in cattle. Nauchnye Tr Nauchno-Issledovatel’skogo Vet Instituta, Minsk. 1970;8: 31–37.

Bachvarova Y., Kostov G., Lilkova N., Savova S., Bardarov I., Baitchev Z. Occurrence of the non-specific tuberculin reactions in cattle and nature of the accompanying pathomorphological and bacterial findings. Bulg J Vet Med. 1999;2: 33–39.

Belloli A, Agosti M, Proverbio D, Avezza F. Interference of bovine Fasciola hepatica on the tuberculin intradermal test reaction. Bov Pract. 1995;0: 141–142.

Bratanovic UE. UK. Bull l’Office Int des Epizoot. 1954;42: 278.

Brito R, Perez Y, Cotrina N. [Correlation of fasciola Hepatica findings in animals reacting to the tuberculin test [bovines]]. 1 National Day of the Cuban Society of Epizootiology and Anthropozoonosis, La Habana (Cuba), 23-24 Nov 1979. Centro de Informacion y Documentacion Agropecuario; 1979. Available: <http://agris.fao.org/agris-search/search.do?recordID=CU8000553>

El-Ahwal AMA. [Effect of experimental fascioliasis on the results of the intradermal tuberculin test in the guinea pig]. Berliner und Münchener tierärztliche Wochenschrift. 1969;82: 484–5.

Feldjusin W. No Title. Veterinarija. 1952;29: 32.

Hartwigt H, El-Ahwal AMA. Untersuchungen ueber die bedeutung der Fasciolose als Ursache positiver Tuberkulinreaktionen beim Rind. Berliner und Muenchener Tieraerztliche Wochenschrift1. 1968;81: 315–316.

Hejj L, Nyiredy I, Tuboly S. Role of Fasciola hepatica in inducin tuberculin allergy in cattle. Zentralblatt fur Bakteriol Parasitenkd Infekt und Hyg. 1969;210: 387-.

Keller H. Relationship between tuberculosis of the liver and liver flukes. [Internet]. Parasites, Vectors, Pathogens and Biogenic Diseases of Animals. 1952.

Kochnev PN. Fasciola in the lungs of cattle. Veterinariya. 1950;27.

Kokurichev PI, Karabainov MA. Specificity of the tuberculin test in cattle with fascioliasis. Sb Nauchnikh Tr Leningr Inst Usovershenstvovaniya Vet Vrachei. 1957;11: 81–85.

Ljesevic Z. Effect of parasitic allergy on the tuberculin test in cattle. Acta Vet Brno. Belgrade; 1957;7: 81–91.

Losieczka K. Periodic occurrence of non-specific tuberculin reactions in cattle. Med Weter. 1960;16: 720–723.

Manukyan ZK. Non-specific tuberculin reactions in cattle with fascioliasis. Tr Armyanskogo Nauchno-Issledovatelskogo Vet Instituta. 1955;8: 25–28.

Merlen H. F. hepática and the tuberculin reaction. Vet Rec. 1950;62.

Nyiredy I, HeJj L, Tuboly S. The role of saprophytic mycobacteria in inducing tuberculin sensitivity in cattle. Magy Allatorvosok Lapja. 1966;21: 433–439.

Parra Florez AD. Prevalencia de la tuberculosis bovina en la sabana de Bogota como base para un programa de control y erradicacion. Programa Univ. Nacional de Colombia. 1982.

Paterson AB. The tuberculin test in tuberculosis-free cattle. Proc R Soc Med. Royal Society of Medicine Press; 1957;50: 253–5.

Piñeiro-Pérez R, García-Hortelano M, José Mellado M, García-Ascaso M, Medina-Claros A, Fernández N, et al. Is there interference in the interpretation of the tuberculin skin test in children with intestinal parasitic infestation? Pediatr Infect Trop Dis Unit, Dep Pediatr Hosp Carlos III, Madrid, Spain. Taylor & Francis; 2012;106: 172–6. doi:10.1179/2047773212Y.0000000023

Quarante. Les reactions parasitaires distomiennes en interference aved les reactions tuberculiniques (intra-dermo reactions). Rev PathComp Med Exp. 1970;70: 29.

Soulsby EJL. Skin hypersensitivity in cattle infested with Fasciola hepatica. J Comp Pathol Ther. Elsevier; 1954;64: 267–274.

Strelchenok K. Non-specificity of tuberculin reactors in cattle with fascioliasis. Veterinarija. 1953;30: 26.

Thompson RCA, Howell MJ. Effect of BCG on the resistance of rats to infection with Fasciola hepatica. Zeitschrift fur Parasitenkd Parasitol Res. 1979;61: 93–98. doi:10.1007/BF00927090

Yamaguchi M, Azuma R, Kawanishi Y. Studies on no visible lesion reactor 1. Correlation of fasciolaiasis to tuberculin rection. Japanese J Vet Sci. 1955;17: 133–137.

Zorawski H, Lipiec M, Skwarek P, Cegielski A. Occurence of acid-fast bacilli in the liver of cattle infected with Fasciola hepatica. Bull Vet Inst Pulawy. 1987;30–31: 1–5
